# *STK11* and DNA Repair Gene Mutations Define Hereditary Subset of Middle Eastern Papillary Thyroid Cancer

**DOI:** 10.3390/ijms27062656

**Published:** 2026-03-14

**Authors:** Rong Bu, Wael Haqawi, Eman A. Abdul Razzaq, Saud Azam, Kaleem Iqbal, Zeeshan Qadri, Sandeep Kumar Parvathareddy, Maha Alrasheed, Khadija Alobaisi, Fouad Al-Dayel, Abdul Khalid Siraj, Khawla S. Al-Kuraya

**Affiliations:** 1 Genomic Analytics, Research Laboratories, Innovation and Research, King Faisal Specialist Hospital and Research Centre, Riyadh 11211, Saudi Arabia; rbu@kfshrc.edu.sa (R.B.); whaqawi@kfshrc.edu.sa (W.H.); miqbal@kfshrc.edu.sa (K.I.); 2 Translational Oncology Research, Research Laboratories, Innovation and Research, King Faisal Specialist Hospital and Research Centre, Riyadh 11211, Saudi Arabia; eabdulrazaq@kfshrc.edu.sa (E.A.A.R.); kalobaisi@kfshrc.edu.sa (K.A.); 3 Human Cancer Genomic Research, Research Laboratories, Innovation and Research, King Faisal Specialist Hospital and Research Centre, Riyadh 11211, Saudi Arabiasqadri96@kfshrc.edu.sa (Z.Q.); psandeepkumar@kfshrc.edu.sa (S.K.P.); mrasheed@kfshrc.edu.sa (M.A.); 4 Anatomic Pathology Department, King Faisal Specialist Hospital and Research Centre, Riyadh 11211, Saudi Arabia; dayelf@kfshrc.edu.sa

**Keywords:** papillary thyroid cancer, germline mutations, cancer predisposition genes, DNA repair genes, exome sequencing, Saudi Arabia

## Abstract

Papillary thyroid cancer (PTC) is the most common endocrine malignancy with especially high incidence in Middle Eastern populations. While classical hereditary syndromes explain a minority of cases, the broader germline landscape of non-syndromic PTC remains unclear. Whole-exome sequencing was performed on 245 unselected Saudi PTC patients to identify germline pathogenic or likely pathogenic variants (PVs/LPVs) in cancer predisposition genes. Clinical and molecular characteristics, and family history were integrated to assess phenotypic correlations. Eleven patients (4.5%) harbored germline PVs/LPVs in cancer susceptibility genes including *STK11*, *TP53*, *BRCA1*, *BRCA2*, *FANCA*, *SLX4*, *RAD50*, *MSH6*, *POLD1* and *NF1*. Four patients (36.4%) carried PVs/LPVs in canonical FA pathway genes; this increased to five patients (45.5%) when *RAD50* was included. Two unrelated patients harbored the same *STK11* variant (p.R304Q) without classical Peutz–Jeghers syndrome features. A *TP53* hotspot mutation (p.R175H) was identified in a patient with a personal history of gastric cancer, a malignancy associated with Li–Fraumeni syndrome. Notably, the *BRCA1* PV detected matches a known Saudi founder mutation in hereditary breast cancer, now observed in PTC. Most germline positive cases lacked syndromic manifestations, underscoring limitations of phenotype or family history-driven genetic testing strategies. These findings suggest that a small subset of non-syndromic PTC cases may carry germline PVs/LPVs in cancer predisposition genes, highlighting the need for broader genetic screening frameworks. Unbiased whole-exome analysis in unselected cohorts can uncover under-recognized genetic risk and guide screening strategies to address the unique hereditary landscape of thyroid cancer in underrepresented populations.

## 1. Introduction

Papillary thyroid cancer (PTC) is the most common histologic subtype of thyroid cancer, comprising approximately 80–90% of all cases globally [1]. Its incidence has been rising steadily over the past two decades [2], with particularly high rates observed in Middle Eastern countries such as Saudi Arabia where thyroid cancer now ranks the second most common cancer among women [3,4].

While the global increase in PTC incidence is partly attributed to advances in imaging and diagnostic sensitivity, emerging evidence suggests that underlying biological and genetic factors also contribute to its rising burden [5,6]. In this context, understanding the contribution of inherited genetic factors has become increasingly important.

Although most cases of PTC are sporadic, inherited predisposition is estimated to account for approximately 5–10% of non-medullary thyroid cancer (NMTC) [7,8,9,10]. These hereditary cases may occur either as part of well-defined cancer syndromes or in non-syndromic familial settings.

Syndromic diseases such as Cowden syndrome (linked to *PTEN*) [11,12], DICER syndrome, familial adenomatous polyposis (*APC*) [13] and Li–Fraumeni syndrome (*TP53*) [14] have been well characterized. However, the genetic basis of non-syndromic PTC remains poorly defined with no single high-penetrance gene identified as a dominant driver.

Furthermore, most prior studies have been limited by small sample sizes, targeted gene panels, or selection biases toward patients with strong family histories or syndromic features. Consequently, the true germline contribution to non-syndromic PTC, particularly in underrepresented populations, remains uncertain.

Recent efforts have begun to explore the broader landscape of germline cancer predisposition in PTC using next generation sequencing platforms [15,16].

These studies suggest that pathogenic or likely pathogenic variants (PVs/LPVs) in genes related to DNA repair, tumor suppression and cell cycle regulation may contribute to thyroid tumorigenesis even in the absence of classical syndromic phenotype [17,18,19]. However, such data remain sparse in Middle Eastern populations, where population-specific founder effects may further shape the germline mutational spectrum [20].

To address these gaps, and provide a more comprehensive assessment of hereditary susceptibility, we conducted germline whole-exome sequencing on a cohort of 245 unselected patients with PTC from Saudi Arabia. Our objective was to identify clinically relevant germline PVs/LPVs across the full spectrum of established cancer susceptibility genes, regardless of syndromic features or family history.

By integrating this unbiased genetic approach with clinical and demographic data, we aim to expand the understanding of hereditary contributions to PTC and inform future strategies for genetic screening and risk assessment, particularly in underrepresented populations.

## 2. Results

### 2.1. Clinico-Pathological Characteristics of the Study Cohort

The median age in this group was 39.2 years with patients ranging from 10 to 83 years, and the male to female ratio was 1 to 3.6. Most tumors were of the classical type making up 50.2% (123/245). Multifocal tumors were seen in 47.8% (117/245) of patients. Extrathyroidal extension was found in 41.6% (102 out of 245), while regional lymph node metastasis was noted in 38.4% (94/245) of cases. At the time of diagnosis, distant metastasis was identified in 6.5% (16/245) (Table 1).

### 2.2. Identification of PVs/LPVs in the Cohort

In the entire cohort, 12 different types of germline PVs/LPVs were identified in cancer susceptibility genes among 11 cases (4.5%), including *STK11*, *TP53*, *BRCA1*, *BRCA2*, *FANCA*, *SLX4*, *RAD50*, *MSH6*, *POLD1* and *NF1* (Table 2). This corresponds to 13 PV/LPVs occurrences across 11 cases because the *STK11* variant occurred in two cases and one case carried two *SLX4* variants. All detected variants were heterozygous. Four patients (36.4%) carried PVs/LPVs in canonical FA pathway genes; this increased to five patients (45.5%) when *RAD50* was included. Eight patients (72.7%) harbored ten PVs/LPVs in eight DNA repair genes, defined here as genes involved in DNA repair and genome integrity, including FA/homologous recombination/mismatch repair/double-strand break repair pathways and *TP53*-mediated DNA damage response, as detailed in Table 2. All identified variants have been previously reported in public databases such as ClinVar and/or in the published literature. Variant classifications were independently evaluated according to American College of Medical Genetics and Genomics and the Association for Molecular Pathology (ACMG/AMP) guidelines and were consistent with available database annotations at the time of analysis. Clinicopathological characteristics of PTC patients with PVs/LPVs are included in Table 3.

One recurrent missense germline LPV (p.R304Q) of *STK11* was detected in two cases, accounting for 18.2% of all mutant cases and 0.8% of all sequenced cases. A loss of heterozygosity (LOH) analysis on sequencing data of samples derived from tumor tissue of these two cases were performed; however, no LOH/second hit was detected in either tumor. One of these two cases had a positive family history of thyroid cancer (Table 3).

A haplotype analysis was carried out for two patients who had the recurrent variant and sufficient DNA sample using the PHASE version 2.1.1 tool. The analysis showed that the two individuals carrying the mutation c.911G>A; p.R304Q in *STK11* gene shared the same haplotype in a very short region of ~0.64 MB, suggesting that this recurrent mutation has insufficient evidence to be a potential founder.

## 3. Discussion

The germline genetic landscape of PTC, the predominant form of Differentiated Thyroid Cancer (DTC), remains insufficiently explored, particularly in non-syndromic, unselected patients.

Although syndromic forms of NMTC such as Cowden syndrome, FAP, DICER syndrome and Li–Fraumeni syndrome have been well described, the genetic basis of non-syndromic PTC remains largely undefined especially in the Middle Eastern population.

Our study addresses this gap by applying WES analysis to an unselected cohort of 245 Saudi PTC patients, systematically screening across known cancer susceptibility genes without preselection based on clinical features.

PVs/LPVs were identified in 11/245 (4.5%) of patients. Analysis reveals enrichment within the PV/LPV-positive subset of DNA repair genes alterations, particularly those associated with the Fanconi Anemia (FA) and double-strand break (DSB) repair pathways. Our findings suggest *STK11* as a potential candidate susceptibility gene in non-syndromic papillary thyroid carcinoma; however, these observations are hypothesis-generating and warrant validation in larger cohorts and functional studies.

Among 11 patients harboring germline PVs/LPVs, four (36.4%) were involved in canonical FA pathway genes, including *SLX4* (*FANP*), *BRCA1* (*FANCS*), *BRCA2* (*FANCD1*), and *FANCA*. When *RAD50*, a gene functionally linked to the FA network via its role in homologous recombination [21] is included, the proportion rises to five cases (45.5%). These findings suggest the disruption of the FA pathway may represent a previously underappreciated mechanism of genetic susceptibility in PTC.

While prior pan-cancer germline analysis, such as the study by Zhang et al. [22], identified occasional FA pathway mutations in pediatric malignancies, these genes have not been specifically implicated in thyroid cancer. Similarly, *SLX4* mutations have been described in FA subtype P [23] and in rare cases of breast and ovarian cancer, but not previously in PTC [24].

The recurrence of *SLX4* mutations in two unrelated patients suggests potential allelic significance and expands the candidate gene landscape for hereditary thyroid cancer.

Although the majority of patients in our cohort did not have a documented family history of PTC, germline PVs/LPVs were identified in 4.5% of the cases.

The detection of these variants in largely non-familial presentations underscores the limitations of relying solely on family history for hereditary risk assessments and suggests that a meaningful proportion of germline predisposition may be attributable to de novo or low-penetrance variants.

Our findings demonstrating a substantial fraction of PVs/LPVs in DNA repair genes such as *FANCA* and *RAD50*, are consistent with emerging evidence linking impaired DNA repair capacity to thyroid carcinogenesis. Qin et al. 2021 [25] reported that patients with papillary thyroid microcarcinoma (PTMC) and reduced double-strand break (DSB) repair capacity had significantly increased risk of developing thyroid cancer, particularly following radiation exposure. Notably, *FANCA* and *RAD50* were among the most frequently mutated genes in their cohort, mirroring our germline findings. Together, these observations reinforce the biological plausibility that genomic instability due to DNA repair pathways may contribute to PTC development, even in the absence of classical syndromic features.

Equally noteworthy is our identification of germline *STK11* mutations in two of 245 PTC cases (0.8%), both carrying the same variant (c.911G>A; p.R304Q). Neither patient exhibited classical features of Peutz–Jeghers syndrome (PTS). One patient had a family history of T-cell lymphoma and family history of PTC (grandmother). Both variants were non-truncating and lacked detectable somatic second hits or loss of heterozygosity (LOH).

A haplotype analysis was performed for the two unrelated patients carrying the recurrent *STK11* p.R304Q germline variant to investigate a possible founder effect. The analysis revealed a shared haplotype block of approximately 0.64 Mb, which is relatively small. Therefore, there is insufficient evidence to establish a potential founder effect for this mutation.

The co-occurrence of thyroid cancer, T-cell lymphoma and family history in the absence of PJS features suggests a non-syndromic, atypical/non-classical *STK11*-associated presentation.

Similarly, a *TP53* hotspot mutation (p.R175H) was identified in a PTC patient with personal cancer history of early onset stomach cancer, a malignancy with the extended Li–Fraumeni syndrome spectrum. Although patients did not meet full clinical criteria for the syndrome, this finding suggests an atypical expression of *TP53*-related cancer predisposition and underscores the phenotype variability associated with germline TP53 mutations [26].

Additionally, the *BRCA* mutation identified in our cohort is a known founder variant in Saudi population [27,28]. Its presence in patients with non-syndromic PTC reinforces the hypothesis that population-specific founder mutations may extend their oncogenic impact across multiple organ sites. These observations further validate the importance of incorporating founder effect data into germline testing strategies in underrepresented populations.

Interestingly, one patient in our cohort harbored concurrent PV/LPV in *SLX4* and *BRCA2*, two critical components of the homologous recombination and FA repair pathway. This co-mutation may represent compounding defects in DNA repair fidelity, potentially resulting in heightened genomic instability and elevated cancer susceptibility. The presence of dual germline hits despite the absence of overt syndrome features emphasizes the need to consider synergistic effects within the same molecular pathway when evaluating hereditary risk and highlights the relevance of pathway-level analyses beyond gene screening in future precision oncology approaches.

Collectively, Our findings reveal that a substantial fraction of germline PV/LPV-positive PTC cases are attributable to mutations in DNA repair genes, particularly within FA and DSB repair pathways. This contrasts with heterogeneous results of previous studies, where no dominant high-penetrance gene has been identified. Although FNMTC accounts for approximately 5% of NMTC cases, our unbiased exome-wide screening uncovered a substantial fraction of PV/LPVs, suggesting gene-based testing may uncover hereditary predisposition even without overt familial clustering.

Beyond FA and DSB repair, the identification of germline *MSH6* and *POLD1* mutations underscores a broader role of mismatch repair and replication fidelity defects in PTC susceptibility. Although infrequent, these mutations are clinically actionable and support consideration of broader genetic strategies particularly in selected patient populations.

This study is primarily descriptive and does not include population-matched controls or enrichment/burden analyses; therefore, the observed PV/LPV patterns should be interpreted as hypothesis-generating. A minority of germline DNA samples were derived from histologically normal FFPE tissue (19 cases; 1 PV/LPV-positive), which may introduce FFPE-associated technical variability; however, histologically normal tissue was independently reviewed and marked by two pathologists with tumor areas strictly excluded. Furthermore, LOH analysis was feasible only in a limited number of STK11 carriers with available paired tumor material, which restricts interpretability and generalizability; these findings are therefore descriptive. The absence of orthogonal validation is acknowledged as a limitation. The haplotype analysis is exploratory due to the limited number of carriers and should be interpreted cautiously. Formal PCA-based ancestry inference and computational contamination estimation were not performed, as the cohort is clinically homogeneous and the study is descriptive without population-comparative analyses. Contamination control relied on histologic review of normal tissue and standard germline DNA extraction procedures. These aspects are acknowledged as limitations.

In summary, our study highlights the identification of germline PVs/LPVs particularly in *STK11*, *TP53*, *SLX4*, *FANCA*, *BRCA1/2* and *RAD50* defined previously under recognized hereditary subset of PTC. These findings support incorporating DNA repair, founder genes into germline testing strategies, and underscore the need for broader population specific approaches to risk stratification and surveillance.

Future research should aim to validate these observations in larger cohorts across different ethnic groups to assess penetrance and improve early detection and surveillance in genetically predisposed individuals

## 4. Materials and Methods

### 4.1. Study Cohort

Clinical samples from 245 patients diagnosed with PTC between 1990 and 2020 at King Faisal Specialist Hospital and Research Center (KFSHRC) were included. Clinicopathological details were collected from medical records and presented in Table 1. All 245 diagnosed PTC cases included in this cohort underwent germline whole-exome sequencing and passed basic quality control, with no samples excluded after QC. A study flow diagram summarizing the 245 case cohort has been presented as Supplementary Figure S1.

This study received ethical approval from the Institutional Review Board and the Research Advisory Council (RAC) of KFSHRC (approval number: 2110 031; approval date: 6 February 2012). Written informed consent was obtained from all participants prior to inclusion in the study.

### 4.2. DNA Extraction and Library Preparation

Genomic DNA extraction was performed using QIAGEN Gentra kit (Minneapolis, MN, USA) as described previously [29]. Germline DNA was primarily extracted from peripheral blood (*n* = 226). Normal formalin-fixed paraffin-embedded (FFPE) tissue was used in a minority of cases (*n* = 19). Among the 11 PV/LPV-positive patients, germline DNA was derived from blood in 10 cases and from normal FFPE tissue in one case. For FFPE-derived germline samples, histologically normal tissue was independently reviewed and marked by two pathologists prior to DNA extraction, with tumor areas strictly excluded. Samples that met quality control (QC) standards proceeded to library construction.

Standard exome capture libraries were generated using the Agilent SureSelectXT Target Enrichment kit, Santa Clara, CA, USA. The library preparation process involved random fragmentation of the DNA, followed by 5′ and 3′ adapter ligation. The adapter-ligated fragments were then amplified via PCR. Subsequently, the prepared library was loaded onto a flow cell where fragments were captured by surface-bound oligonucleotides complementary to the library adapters. Each fragment was then amplified into a distinct clonal cluster through bridge amplification, preparing the templates for sequencing.

### 4.3. Whole-Exome Sequencing

Sequencing was performed on the Illumina NovaSeq 6000 platform, San Diego, CA, USA. Raw image data was processed into base calls, generating binary base call (BCL) files. These BCL files were subsequently de-multiplexed and converted to the FASTQ format using Illumina’s bcl2fastq software (v2.20.0). The quality of the sequencing reads in the FASTQ files was assessed using FastQC (v0.11.9).

### 4.4. Data Analysis and Variant Calling

Sequencing reads were aligned to the human reference genome (hg19) using the Burrows–Wheeler Aligner (BWA, v0.7.15). Alignment to hg19 was performed to maintain compatibility with the institution’s validated analysis pipeline and legacy annotation resources used for this cohort. Sequencing yielded a median read depth of 218× (range: 93–1008×) per sample. Post-alignment processing, including local realignment and marking of PCR duplicates, was performed using Picard tools (v1.119). Base quality score recalibration was conducted with the Genome Analysis Toolkit (GATK, v4.0.12.0).

Variant calling was performed on each sample using the HaplotypeCaller tool within GATK package (v4.0.12.0). The identified variants were subsequently annotated using the ANNOVAR tool (available at: http://annovar.openbioinformatics.org/; accessed on 12 January 2024) [30]. Variant annotations were gathered from multiple publicly available genomic databases, including dbSNP138, the 1000 Genomes Project, ESP6500, the Exome Aggregation Consortium (ExAC), ClinVar, and other related resources. Germline variants were filtered using a minimum read depth of ≥20, requiring at least five supporting alternate reads. Heterozygous variants were accepted only if the variant allele fraction (VAF) fell between 0.30 and 0.85. All reported PV/LPVs were manually reviewed in IGV to confirm read alignment and exclude sequencing artifacts, such as strand bias or localization at the ends of reads. Population frequency annotation and variant validation were performed using gnomAD v2.1 (hg19 build) along with ExAC, dbSNP build 138, and ClinVar where applicable. Furthermore, variants with more than two reads exhibiting a mapping quality of zero were excluded as false positives. The classification of variants as pathogenic or likely pathogenic followed the established guidelines set forth by the American College of Medical Genetics and Genomics and the Association of Molecular Pathology (ACMG/AMP) [31]. Orthogonal validation by Sanger sequencing was not performed. All reported PV/LPVs were supported by adequate sequencing depth and manual IGV review.

### 4.5. LOH Analysis

Loss of heterozygosity (LOH) of the *STK11* germline pathogenic variants was evaluated using paired tumor–normal DNA sequencing data. Paired tumor–normal WES data was available for *n* = 2 *STK11* carriers (one tumor per carrier), utilizing a minimum threshold of five alternative reads and a regional sequencing depth of ≥20. LOH at the variant locus was defined as a shift in allelic status from heterozygous in the germline sample to either homozygous or hemizygous in the matched tumor sample, based on comparative variant calling between the paired datasets. 

### 4.6. Haplotype Analysis

A high-throughput SNP genotyping array (Illumina Infinium) was employed for genotyping, utilizing a custom design panel of 778,783 SNPs, according to the manufacturer’s instructions (Illumina Inc., San Diego, CA, USA). Genotype calls and normalized signal intensities were determined with the aid of the Illumina Bead Array Files Python library (available at: https://github.com/Illumina/BeadArrayFiles; accessed on 2 June 2024). The quality assessment included monitoring p10 GC scores and sample call rates. The resulting filtered genotype data for all analyzed samples and probes were compiled into a text file. Additionally, SNP data for 100 control subjects were sourced from our in-house database.

We performed haplotype reconstruction for the two test samples alongside the 100 control individuals using tool PHASE (version 2.1.1) [32,33]. Input for the algorithm consisted of variant and nucleotide positions, along with the genotypes for all subjects at those loci. The analysis was run with 100 iterations, a thinning interval of one, and a burn-in of 100. Haplotype analysis was performed as an exploratory assessment to evaluate whether the recurrent STK11 variant may share a common haplotype background.

## Figures and Tables

**Table 1:** Clinicopathological variables for the patient cohort (*n* = 245).

	Total	
	No.	%
**Total**	245	
Age at surgery (years)		
Median (range)	39.2 (10.0–83.0)	
<55	207	84.5
≥55	38	15.5
Gender		
Male	53	21.6
Female	192	78.4
Histologic subtype		
Classical variant	123	50.2
Follicular variant	52	21.2
Tall cell variant	36	14.7
Other variants	34	13.9
Tumor laterality		
Unilateral	177	72.2
Bilateral	68	27.8
Tumor focality		
Unifocal	128	52.2
Multifocal	117	47.8
Extrathyroidal extension		
Absent	143	58.4
Present	102	41.6
Lymphovascular invasion		
Present	94	38.4
Absent	151	61.6
T stage		
T1	61	25.0
T2	54	22.0
T3	114	46.5
T4	16	6.5
Regional LN metastasis		
N0	116	47.3
N1	94	38.4
Nx	35	14.3
Distant metastasis		
Present	16	6.5
Absent	229	93.5
TNM Stage		
I	214	87.3
II	20	8.2
III	6	2.5
IV	5	2.0

LN, lymph node; TNM, tumor–node–metastasis staging system; T, primary tumor status; N, regional lymph node status; Nx, regional lymph nodes not assessed.

**Table 2:** Germline pathogenic and likely pathogenic variants identified in papillary thyroid carcinoma cases.

S No.	Gene	Mutation	Type of Mutation
1	*STK11*	c.911G>A; p.R304Q	Missense
2	*STK11*	c.911G>A; p.R304Q	Missense
3	*SLX4*	c.4409delC; p.P1470Rfs*37	Frameshift
		c.4394_4404del; p.R1465Lfs*33	Frameshift
4	*SLX4*	c.1346dup; p.R450Efs*9	Frameshift
	*BRCA2*	c.4419_4423dup; p.M1475Tfs*6	Frameshift
5	*BRCA1*	c.4136_4137del; p.S1379*	Stopgain
6	*TP53*	c.524G>A; p.R175H	Missense
7	*MSH6*	c.1518dup; p.R507X	Stopgain
8	*POLD1*	c.1006C>T; p.Q336X	Stopgain
9	*RAD50*	c.412C>T; p.R138X	Stopgain
10	*NF1*	c.2842C>T; p.Q948X	Stopgain
11	*FANCA*	c.3761_3762del; p.E1254Gfs*23	Frameshift

* indicates a translation termination codon, consistent with HGVS nomenclature.

**Table 3:** Clinicopathological characteristics of PTC cases with PVs/LPVs.

S No	AGE	Gender	Laterality	Focality	ETE	LVI	pT	pN	pM	Stage	BRAF	KRAS	Family History	Gene	Mutation	Alive/Dead	Presence of Other Cancer
1	25	Female	Unilateral	Unifocal	Absent	Absent	T2	N0	M0	I	No	No	Yes (Grandmother—thyroid carcinoma)	*STK11*	c.911G>A; p.R304Q	Alive	Cutaneous T-cell lymphoma
2	26	Female	Unilateral	Unifocal	Absent	Absent	T2	N0	M0	I	Yes	No	No	*STK11*	c.911G>A; p.R304Q	Alive	No
3	36	Male	Unilateral	Unifocal	Present	Absent	T2	N1b	M0	I	Yes	No	No	*SLX4*	c.4409delC; p.P1470Rfs*37	Alive	No
															c.4394_4404del; p.R1465Lfs*33		
4	54	Male	Unilateral	Multifocal	Present	Present	T2	N0	M0	I	Yes	No	No	*SLX4*	c.1346dup; p.R450Efs*9	Alive	No
														*BRCA2*	c.4419_4423dup; p.M1475Tfs*6		
5	64	Female	Unilateral	Multifocal	Present	Present	T2	N0	M0	II	Yes	No	No	*BRCA1*	c.4136_4137del; p.S1379*	Alive	No
6	38	Female	Unilateral	Unifocal	Absent	Absent	T1	N0	M0	I	No	No	No	*TP53*	c.524G>A; p.R175H	Alive	Gastric cancer
7	67	Female	Bilateral	Multifocal	Absent	Present	T1	N0	M0	I	No	No	No	*MSH6*	c.1518dup; p.R507X	Alive	No
8	36	Female	Bilateral	Multifocal	Absent	Absent	T1	N1a	M0	I	Yes	No	No	*POLD1*	c.1006C>T; p.Q336X	Alive	No
9	28	Female	Unilateral	Unifocal	Absent	Absent	T2	N1a	M0	I	No	No	No	*RAD50*	c.412C>T; p.R138X	Alive	No
10	36	Female	Unilateral	Unifocal	Absent	Absent	T1	N0	M0	I	No	No	No	*NF1*	c.2842C>T; p.Q948X	Alive	No
11	58	Male	Unilateral	Unifocal	Absent	Absent	T3	N0	M1	IV	No	No	No	*FANCA*	c.3761_3762del; p.E1254Gfs*23	Alive	No

* indicates a translation termination codon, consistent with HGVS nomenclature. ETE, extrathyroidal extension; LVI, lymphovascular invasion; pT, pathological primary tumor stage; pN, pathological regional lymph node stage; pM, pathological distant metastasis stage.

## Data Availability

The original contributions presented in the study are included in the article; further inquiries can be directed to the corresponding authors.

## Supplementary Materials

The following supporting information can be downloaded at: https://www.mdpi.com/article/10.3390/ijms27062656/s1.

## References

[1] Lloyd R.V., Buehler D., Khanafshar E. (2011). Papillary thyroid carcinoma variants. Head. Neck Pathol..

[2] Pellegriti G., Frasca F., Regalbuto C., Squatrito S., Vigneri R. (2013). Worldwide Increasing Incidence of Thyroid Cancer: Update on Epidemiology and Risk Factors. J. Cancer Epidemiol..

[3] Hussain F., Iqbal S., Mehmood A., Bazarbashi S., ElHassan T., Chaudhri N. (2013). Incidence of thyroid cancer in the Kingdom of Saudi Arabia, 2000–2010. Hematol. Oncol. Stem Cell Ther..

[4] Qusty N.F., Albarakati A.J.A., Almasary M., Alsalamah S., Alharbi L., Alharthi A., Al Sulaiman I.N., Baokbah T.A.S., Taha M. (2023). Thyroid Cancer Knowledge and Awareness in Saudi Arabia: A Cross-Sectional Study. Cureus.

[5] Rangel-Pozzo A., Sisdelli L., Cordioli M.I.V., Vaisman F., Caria P., Mai S., Cerutti J.M. (2020). Genetic Landscape of Papillary Thyroid Carcinoma and Nuclear Architecture: An Overview Comparing Pediatric and Adult Populations. Cancers.

[6] Mussazhanova Z., Rogounovitch T.I., Saenko V.A., Krykpayeva A., Espenbetova M., Azizov B., Kondo H., Matsuda K., Kalmatayeva Z., Issayeva R. (2021). The Contribution of Genetic Variants to the Risk of Papillary Thyroid Carcinoma in the Kazakh Population: Study of Common Single Nucleotide Polymorphisms and Their Clinicopathological Correlations. Front. Endocrinol..

[7] Sippel R.S., Caron N.R., Clark O.H. (2007). An evidence-based approach to familial nonmedullary thyroid cancer: Screening, clinical management, and follow-up. World J. Surg..

[8] Charkes N.D. (2006). On the prevalence of familial nonmedullary thyroid cancer in multiply affected kindreds. Thyroid.

[9] Capezzone M., Robenshtok E., Cantara S., Castagna M.G. (2021). Familial non-medullary thyroid cancer: A critical review. J. Endocrinol. Invest..

[10] Baagar K.A., Alowainati B.I. (2021). Papillary Thyroid Cancer Affecting Multiple Family Members: A Case Report and Literature Review of Familial Nonmedullary Thyroid Cancer. Case Rep. Endocrinol..

[11] Ngeow J., Mester J., Rybicki L.A., Ni Y., Milas M., Eng C. (2011). Incidence and clinical characteristics of thyroid cancer in prospective series of individuals with Cowden and Cowden-like syndrome characterized by germline PTEN, SDH, or KLLN alterations. J. Clin. Endocrinol. Metab..

[12] Ngeow J., Stanuch K., Mester J.L., Barnholtz-Sloan J.S., Eng C. (2014). Second malignant neoplasms in patients with Cowden syndrome with underlying germline PTEN mutations. J. Clin. Oncol..

[13] Soravia C., Sugg S.L., Berk T., Mitri A., Cheng H., Gallinger S., Cohen Z., Asa S.L., Bapat B.V. (1999). Familial adenomatous polyposis-associated thyroid cancer: A clinical, pathological, and molecular genetics study. Am. J. Pathol..

[14] Yang S.P., Ngeow J. (2016). Familial non-medullary thyroid cancer: Unraveling the genetic maze. Endocr. Relat. Cancer.

[15] Yu Y., Dong L., Li D.P., Chuai S.K., Wu Z.G., Zheng X.Q., Cheng Y.A., Han L., Yu J.P., Gao M. (2015). Targeted DNA Sequencing Detects Mutations Related to Susceptibility among Familial Non-medullary Thyroid Cancer. Sci. Rep.

[16] Gara S.K., Jia L., Merino M.J., Agarwal S.K., Zhang L., Cam M., Patel D., Kebebew E. (2015). Germline HABP2 Mutation Causing Familial Nonmedullary Thyroid Cancer. N. Engl. J. Med..

[17] Mio C., Verrienti A., Pecce V., Sponziello M., Damante G. (2021). Rare germline variants in DNA repair-related genes are accountable for papillary thyroid cancer susceptibility. Endocrine.

[18] Kamihara J., Zhou J., LaDuca H., Wassner A.J., Dalton E., Garber J.E., Black M.H. (2022). Germline pathogenic variants in cancer risk genes among patients with thyroid cancer and suspected predisposition. Cancer Med..

[19] Zhao Y., Yu T., Sun J., Wang F., Cheng C., He S., Chen L., Xie D., Fu L., Guan X. (2021). Germ-line mutations in WDR77 predispose to familial papillary thyroid cancer. Proc. Natl. Acad. Sci. USA.

[20] Al Amri W.S., Al Jabri M., Al Abri A., Hughes T.A. (2025). Cancer Genetics in the Arab World. Technol. Cancer Res. Treat..

[21] Michl J., Zimmer J., Tarsounas M. (2016). Interplay between Fanconi anemia and homologous recombination pathways in genome integrity. Embo J..

[22] Zhang J., Walsh M.F., Wu G., Edmonson M.N., Gruber T.A., Easton J., Hedges D., Ma X., Zhou X., Yergeau D.A. (2015). Germline Mutations in Predisposition Genes in Pediatric Cancer. N. Engl. J. Med..

[23] Shah S., Kim Y., Ostrovnaya I., Murali R., Schrader K.A., Lach F.P., Sarrel K., Rau-Murthy R., Hansen N., Zhang L. (2013). Assessment of SLX4 Mutations in Hereditary Breast Cancers. PLoS ONE.

[24] de Garibay G.R., Diaz A., Gavina B., Romero A., Garre P., Vega A., Blanco A., Tosar A., Diez O., Perez-Segura P. (2013). Low prevalence of SLX4 loss-of-function mutations in non-BRCA1/2 breast and/or ovarian cancer families. Eur. J. Hum. Genet..

[25] Qin J., Fan J., Li G., Liu S., Liu Z., Wu Y. (2021). DNA double-strand break repair gene mutation and the risk of papillary thyroid microcarcinoma: A case-control study. Cancer Cell Int..

[26] Bougeard G., Renaux-Petel M., Flaman J.M., Charbonnier C., Fermey P., Belotti M., Gauthier-Villars M., Stoppa-Lyonnet D., Consolino E., Brugieres L. (2015). Revisiting Li-Fraumeni Syndrome From TP53 Mutation Carriers. J. Clin. Oncol..

[27] Siraj A.K., Bu R., Iqbal K., Siraj N., Al-Haqawi W., Al-Badawi I.A., Parvathareddy S.K., Masoodi T., Tulbah A., Al-Dayel F. (2019). Prevalence, spectrum, and founder effect of BRCA1 and BRCA2 mutations in epithelial ovarian cancer from the Middle East. Hum. Mutat..

[28] Bu R., Siraj A.K., Al-Obaisi K.A., Beg S., Al Hazmi M., Ajarim D., Tulbah A., Al-Dayel F., Al-Kuraya K.S. (2016). Identification of novel BRCA founder mutations in Middle Eastern breast cancer patients using capture and Sanger sequencing analysis. Int. J. Cancer.

[29] Abubaker J., Jehan Z., Bavi P., Sultana M., Al-Harbi S., Ibrahim M., Al-Nuaim A., Ahmed M., Amin T., Al-Fehaily M. (2008). Clinicopathological analysis of papillary thyroid cancer with PIK3CA alterations in a Middle Eastern population. J. Clin. Endocrinol. Metab..

[30] Wang K., Li M., Hakonarson H. (2010). ANNOVAR: Functional annotation of genetic variants from high-throughput sequencing data. Nucleic Acids Res..

[31] Richards S., Aziz N., Bale S., Bick D., Das S., Gastier-Foster J., Grody W.W., Hegde M., Lyon E., Spector E. (2015). Standards and guidelines for the interpretation of sequence variants: A joint consensus recommendation of the American College of Medical Genetics and Genomics and the Association for Molecular Pathology. Genet. Med..

[32] Sotiropoulos K., Eleftherakos K., Dzukic G., Kalezic M.L., Legakis A., Polymeni R.M. (2007). Phylogeny and biogeography of the alpine newt Mesotriton alpestris (Salamandridae, Caudata), inferred from mtDNA sequences. Mol. Phylogenet Evol..

[33] Stephens M., Scheet P. (2005). Accounting for decay of linkage disequilibrium in haplotype inference and missing-data imputation. Am. J. Hum. Genet..

