# Supplementary figures and images for: STK11 and DNA Repair Gene Mutations Define Hereditary Subset of Middle Eastern Papillary Thyroid Cancer
Source: Int J Mol Sci. 2026 Mar 14;27(6):2656. doi: 10.3390/ijms27062656 (PMC13026885; doi:10.3390/ijms27062656)

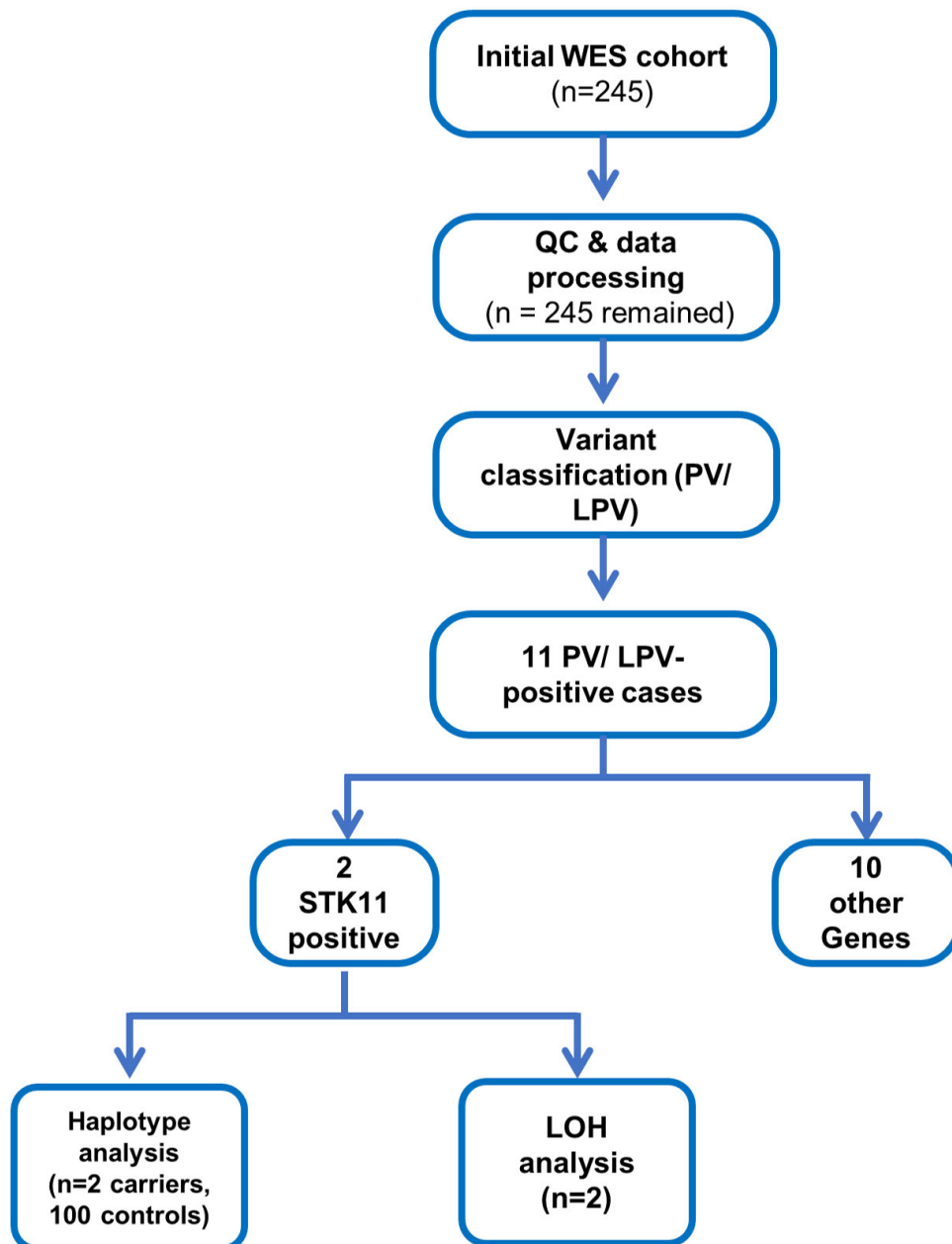

Supplementary Figure S1: Study flow diagram

Supplement: Supplementary file 1 [file ijms-27-02656-s001.zip › ijms-4175055-supplementary.pdf]
